# Epidemics on Networks with Large Initial Conditions or Changing Structure

**DOI:** 10.1371/journal.pone.0101421

**Published:** 2014-07-08

**Authors:** Joel C. Miller

**Affiliations:** School of Mathematical Sciences, School of Biological Sciences, and Monash Academy for Cross & Interdisciplinary Mathematics, Monash University, Melbourne, Victoria, Australia; Imperial College London, United Kingdom

## Abstract

In this paper we extend previous work deriving dynamic equations governing infectious disease spread on networks. The previous work has implicitly assumed that the disease is initialized by an infinitesimally small proportion of the population. Our modifications allow us to account for an arbitrarily large initial proportion infected. This helps resolve an apparent paradox in earlier work whereby the number of susceptible individuals could increase if too many individuals were initially infected. It also helps explain an apparent small deviation that has been observed between simulation and theory. An advantage of this modification is that it allows us to account for changes in the structure or behavior of the population during the epidemic.

## Introduction

The mathematical study of infectious disease spread has contributed significantly to our ability to design effective interventions to reduce disease spread. Most of the earliest models were based on the assumption that disease transmission occurs as a Poisson process and each transmission reaches an individual chosen randomly from the population. This implicitly assumes that partnership duration is very brief. These models have been modified to account for a number of different effects, such as demographic groups [1].

More recently, attempts have been made to incorporate the “network” structure of the population (see, *e.g.*, [2]). Typically these focus on trying to understand the role played by “high-degree” individuals (those individuals with many contacts), and they come in one of two flavors: they either continue the assumption of fleeting partnerships (the timescale of individual transmissions is long compared to the timescale of individual partnerships) [1], [3]–[6], or they take the opposite limit in which the partnership network is static (the timescale of the epidemic is short compared to the timescale of individual partnerships) [7]–[14]. These two approaches do not address the intermediate regimes.

Recent work has shown that for susceptible-infectious-recovered (SIR) models, it is possible to unify these two approaches with an “edge-based compartmental model” (EBCM) that allows partnership duration to range continuously from zero to infinite [15]–[17] [for susceptible-infectious-susceptible (SIS) models, the picture is more complicated, see for example [18]]. The resulting models are low-dimensional and contain many standard models as special cases [17]. Unfortunately, these models are derived under the assumption that the initial proportion infected is infinitesimally small (while the absolute number infected is sufficiently large that the dynamics are deterministic). It is assumed that by the time the equations are used, any early transients have died away. A consequence of this assumption is that the models break down if 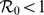 (that is, if the average number of infections caused by an infected individual early in the epidemic is less than 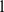) or if the initial proportion infected is not negligible.

The failure if the initial proportion infected is not negligible was observed by [14]. This paper used an early (static network) version of the equations of [15] from [7] and compared them with simulation. A small discrepancy in final sizes was noted. This discrepancy was not present for equations of [19], a system requiring 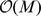 equations where 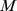 is the maximum degree, or for another system introduced in [14] which required 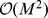 equations.

In the remainder of this paper, we generalize the EBCM equations for the spread of infectious disease assuming a finite proportion of the population is initially infected. We test the resulting equations against simulations, analyze their predictions for different disease scenarios, and investigate the cause of the discrepancies found in previous work. For simplicity we focus on the static network limit. The method we introduce is straightforward to adapt to dynamic networks.

## Analysis

We modify the approach of [15] which assumed an infinitesimal initial proportion infected. We adapt the approach to consider a wide range of possible initial conditions. We assume that the dynamics of the epidemic may be treated as deterministic, which means we assume the population is very large and the initial number infected is large enough for the epidemic to behave deterministically. The assumption that behavior is deterministic may be understood qualitatively as equivalent to the claim that no individual has a large enough effect to alter the dynamics of the disease at the population-scale: the time (or even if) a single given individual becomes infected has a negligible impact on the proportion infected. If stochastic effects are still important but 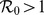, then these equations may become accurate at a later time once sufficient numbers are infected.

We assume the population consists of 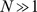 individuals. Each is assigned a degree 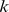 (independently of degrees of other individuals) with probability 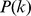 where 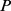 defines a probability distribution on the non-negative integers. The network is wired together using the “Configuration Model” (or “Molloy-Reed”) approach [11], [20]: each individual is assigned a number of stubs (or half-edges) equal to its degree. Pairs of stubs are then wired together to form edges/partnerships. It is likely that this algorithm produces a handful of self-loops or repeated edges, but although they may be present, their density (*i.e.*, the probability a given individual is involved in a self-loop or repeated edge) goes to zero like 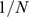.

We define a *test individual* 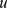 to be a random individual chosen at time 0. Because we assume that the spread is deterministic, this means that the probability 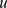 is in a given state is equal to the proportion of the population in that state. So we focus on calculating the probability 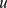 is susceptible, infected, or recovered. We modify 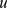 so that it does not transmit to any of its partners if ever infected. This assumption does not affect the probability 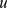 is in any given state, but it does prevent a correlation between the statuses of different partners which would be caused by infection traveling through 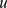. This allows us to treat the partners of 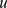 as independent and so each partner of 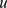 may independently transmit to 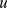. It is important to note that this assumption has no impact on the probability 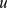 is in any given state and therefore, it does not affect our calculation of the proportion of the population in each state. Further discussion of the test individual is in [21].

### Variables and Parameters

We introduce our variables, our parameters, and their definitions in table 2. The starting point is the test individual 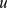. The remaining variables and parameters can be broadly divided into four groups.

**Table 2:** The variables we need to calculate the epidemic dynamics. In all of these 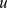 is a test individual: randomly chosen from the population and modified so that it cannot infect others, although it can become infected.

Variable/parameter	Definition
Test Individual 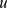	A randomly member of the population chosen at time 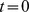 who is prevented from transmitting to its partners.
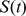	The proportion of the entire population that is susceptible.
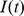	The proportion of the entire population that is infected.
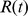	The proportion of the entire population that is recovered.
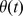	The probability a random partner 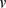 of 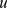 that did not transmit to 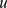 by time 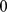 has not transmitted to 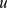 by time 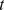 .
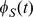	The probability a random partner 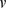 that did not transmit to 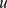 by time 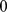 is susceptible at time 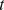 .
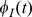	The probability a random partner 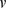 that did not transmit to 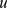 by time 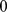 is infected at time 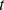 but has not transmitted to 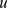 .
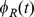	The probability a random partner 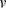 that did not transmit to 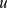 by time 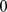 is recovered at time 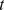 and never transmitted to 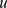 .
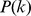	The probability an individual has degree 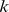 .
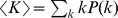	The average degree.
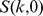	The probability an individual with degree 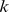 is initially susceptible.
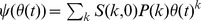	The probability that the test individual 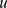 is susceptible at time 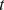 . In a large population this should equal 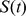 .
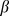	the per-edge transmission rate
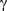	the per-individual recovery rate

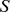
, 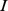, and 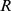 denote the proportion of the population in each state, or equivalently the probability that the test individual 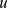 is in each state.

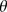
, 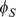, 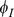, and 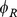 give the probability a partner of 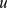 has a given status and the probability the partner has transmitted to 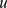: 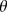 is the probability the partner has not transmitted to 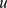 and 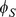, 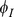, and 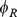 give the probability the partner has not transmitted to 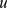 and is susceptible, infected, or recovered respectively.

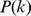
 tells us the probability a random individual has degree 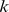, while 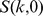 tells us the probability a random individual of degree 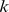 is initially susceptible. The function 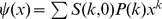 encodes 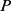 and 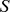. We define 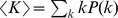 to be the average degree.
- We have two disease parameters to consider: 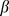, the transmission rate, and 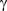, the recovery rate.

Given our definitions, 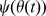 is the probability 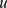 is susceptible at time 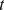. By noting that 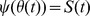, we will be able to close our system of equations.

The main distinction between this approach and the previous EBCM approach [15] is that we use just the initially susceptible individuals to define 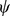 while the earlier work took 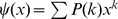, the probability generating function for the degree distribution. The earlier work then assumed the disease had already been spreading prior to time 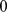 and defined 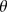 to be the probability that a partner has never transmitted [so 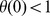] whereas here we take 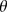 to be the probability that a partner has not transmitted given that it had not prior to time 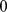 [so 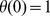].

### Equation Derivation

We will find a closed system of equations based on these variables. We begin by looking at 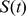. If the test individual 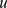 has degree 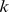 and is susceptible at 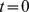, then the probability it is susceptible at some later time is 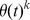. If we do not know 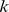 or whether 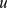 is susceptible at 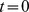, then the probability 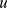 is susceptible at time 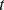 is the sum over all 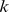 of the product of the probability 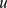 is initially susceptible 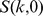 with the probability 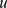 is still susceptible 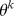. We have 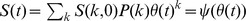. Thus we conclude
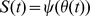



We know that 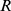 solves 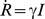. We also have a conservation rule that 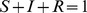, so 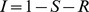. Thus our equations are
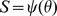





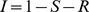





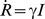



**Figure 1:**
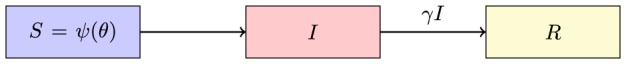
Flow diagram showing the flux of individuals between the different compartments. Because we have an explicit expression for 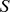, if we know 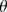 we do not need to explicitly determine the flux from 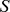 to 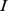.

Assuming 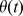 is known, then this system with an initial condition for 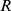 completely defines 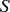, 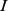, and 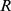. This is shown in the flow diagram in figure 1. Other formulations are possible, for example 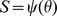, 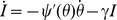, 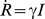. However we find our system to be preferable because it minimizes the number of differential equations.

In order to close this system of equations we need an equation giving 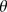. Recall that 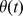 is the probability a partner 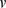 of 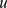 that had not yet transmitted to 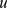 by time 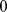 has still not transmitted by time 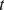. This is broken into three disjoint sub-compartments 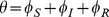 based on whether 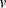 has not transmitted and is susceptible, infected, or recovered. Because 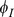 is the probability 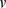 has not transmitted and is infected, it is straightforward to see that 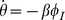. So if we can find 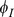 in terms of 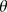, then we arrive at a single equation for 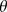, which can be used to provide 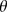 for the 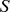, 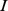, and 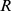 equations.

**Figure 2:**
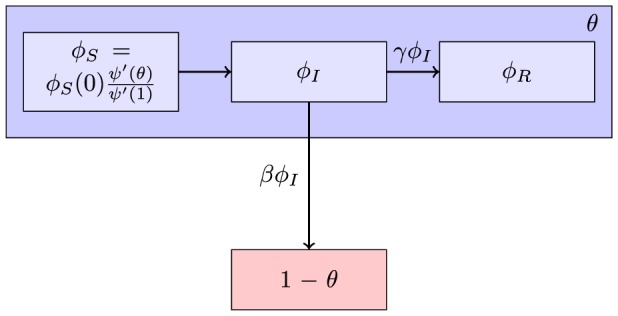
Flow diagram for the flux of partners through different states. The top three boxes 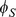, 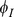, and 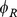 make up 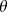 and represent the different states the partner can be in if it has not transmitted. The lower box 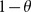 is the probability the partner has transmitted.

To do this, we use the fact that 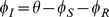 and find 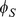 and 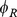 in terms of 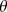. We turn to figure 2. The recovery rate is 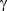 and the transmission rate is 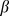, so we have 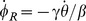. We can integrate this, and using the fact that 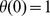 we find 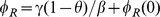. To find 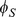 in terms of 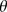, we note that the probability 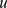 has an edge to a node that is susceptible at time 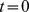 is 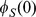. The probability the susceptible partner has degree 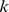 is 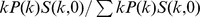, so the probability an initially susceptible partner is susceptible at some later time is 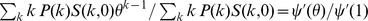. Thus 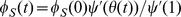. We arrive at
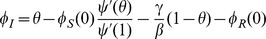
and 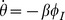 becomes
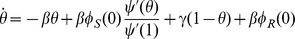
with 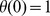. This completes our system.

Our final closed system of equations is
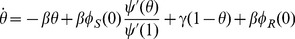
(1)




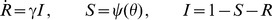
(2)where 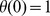, and 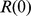 is given by the initial conditions. These equations lead to earlier equations of [7], [15], [22] if 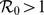 and 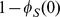, 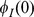, 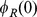 and 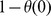 are all infinitesimally small. If 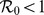, the error caused by the approximations 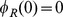 and 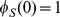 is comparable to the actual number infected.

#### Final size relation

The final size relation assuming small initial condition is well-known [11]. The final size relation for larger initial conditions has recently been found [21] in a more general case not assuming constant transmission and recovery rates. It can be derived easily for this model by setting 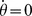. We find
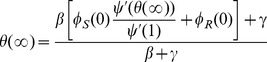
(3)




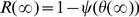
(4)


### Model Validation

We now compare our model with simulations for populations that satisfy the Configuration Model/Molloy-Reed model assumptions. Although an earlier version of these equations was found to have minor discrepancies [14], we show that once we appropriately account for the initial condition, the calculation becomes correct.

#### Final Size Comparison

To show that our new equations accurately calculate the impact of the initial conditions, we first consider epidemic spread in networks with the same degree distribution as in [14] (table 1), but with varying numbers initially infected and varying population sizes. We then consider the impact of selecting high or low degree nodes as the earliest infected individuals, using networks whose degree distributions more clearly show the impact of biased selection of the initial individuals.

**Table 1:** The degree distribution used in simulations in [14].

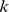	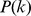
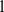	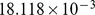
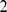	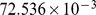
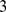	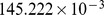
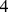	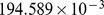
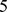	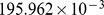
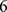	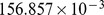
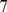	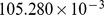
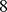	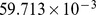
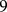	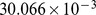
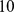	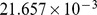

We run a large number of simulations for each number of initial infections. For each simulation we generate a new network. Our simulation technique is similar to those recently described by [8], [19], [23]. In the Configuration Model framework, each node is assigned a degree, nodes are given stubs (or half-edges), and then stubs are randomly paired together. In the simulations we use, each node is assigned a degree, nodes are given stubs, and then the disease begins to spread in the network before stubs are paired. Each time the disease transmits along a stub that stub is joined to a randomly selected as-yet-unpaired stub. If the partner is susceptible, then it becomes infected. If not, nothing happens. Once stubs are paired they remain in their edge. This approach is equivalent to constructing the network in advance and then following the disease, but it is more efficient computationally because it only constructs those parts of the network the disease traces.

#### Randomly selected initial infections

**Figure 3:**
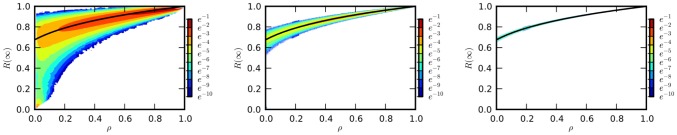
Results of simulations for 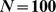, 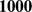, and 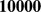 individuals. The solid curve gives our prediction for the final sizes of epidemic in a large population. Colors are log scale giving probability of that particular epidemic size. Each simulation is for a new network generated using the 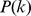 from table 1, with 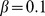 and 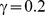. We randomly select a proportion 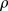 of the population to initially infect and compare final size with the prediction of theory. The number of simulations for each 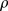 for 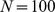, 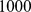, and 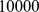 was 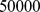, 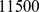, and 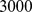 respectively. To show that this is sufficient to resolve the distribution, for 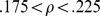 there were 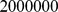, 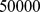 and 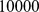 simulations performed respectively for each 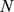. This only slightly improves the tails of the distribution. Note that when the initial number (not proportion) of infections is small, a large fraction of simulations end without an epidemic.

We first consider varying numbers of randomly chosen initial infected individuals. In figure 3 we take the degree distribution from table 1.

We randomly select a proportion 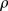 of the population to initially infect. We have 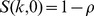 for all 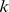, so 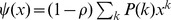. Similarly we have 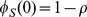. Because the epidemic begins with no recovered individuals, we take 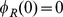. We take 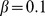 and 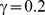 (though all that matters for the final size is their ratio).

We take populations of 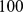, 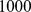, and 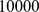 and perform many simulations. We compare the final sizes observed with the final size relation of equations (3) and (4). The equations are derived in the infinite population limit, but in figure 3 we see that even with populations of only 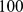 they give a good prediction of the observed behavior. As the population size increases, the distribution becomes narrower and the simulations collapse more tightly around the prediction.

#### Biased initial infections

To show that the approach we have derived can also be applied to cases where the initial infected individuals are selectively chosen based on their degree, we use a different degree distribution that helps highlight the effect. We take 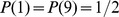. We consider two options. In the first approach, individuals with higher degree are preferentially selected. To do the selection, we choose an individual with probability proportional to the square if its degree, and infect it. We repeat this until a proportion 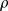 of the population is infected. In the second approach individuals are chosen with probability proportional to the square of their inverse degree until a proportion 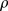 is infected. We take 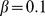 and 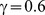.

Using these rules, we clearly see that 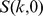 is not uniform. Instead, for the case where individuals are selected with probability proportional to their squared degree, we find that 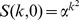 where 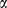 solves 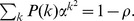 We find 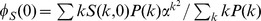. In the case where individuals are selected with probability inversely proportional to their squared degree, we find that 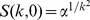 where 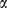 solves 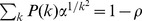, and 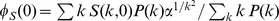.

**Figure 4:**
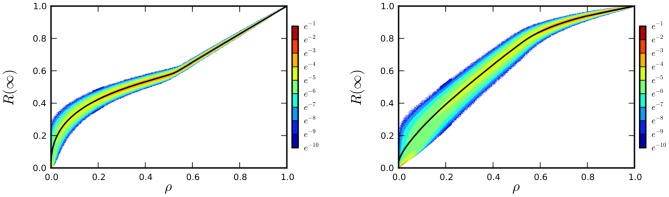
Epidemic final sizes in population of 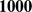 individuals with half having degree 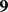 and half with degree 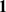. The disease parameters are 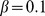, 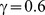. Results of simulations having initial infections chosen with probability proportional to square of degree (left) or inverse square of degree (right). For each initial number of infections, 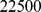 simulations were performed, each with a different network. For the range 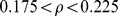, 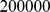 simulations were performed to give insight into how well resolved the distribution is. Note that for small numbers of initial infections, epidemics are less likely when the lower degree individuals are chosen.

We compare predictions and simulations in populations of 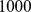 individuals in figure 4. In the limit of a negligible initial proportion infected, the final size of epidemics in these networks is about 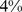. As we increase the number of initially infected individuals, we increase the final size because of these individuals and because of the additional infections they lead to. At small amounts, increasing the initial number of high degree nodes has a much larger impact on the final size because they cause more additional infections. However, as the amount of infection initially present is increased this effect becomes less important: the high degree individuals would become infected anyway. So the largest gain in final size comes from infecting low degree individuals who would not receive an infection from their partners. The “kinks” that occur just above 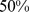 initially infected are because effectively all individuals of high (left) or low (right) degree are initially infected.

#### Dynamic Calculation

**Figure 5:**
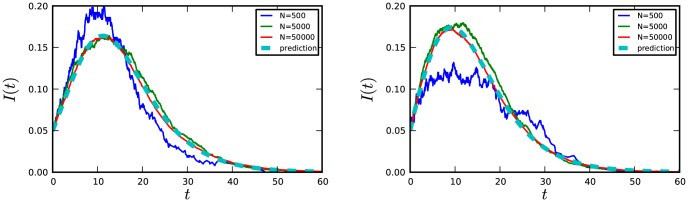
A comparison of the observed and predicted number of infections from simulations. Left: 5% initially infected, chosen randomly from the population. Right: 5% initially infected, chosen with probability proportional to squared degree. Each simulation curve represents a single simulation of the given size. As population size increases, the results converge to the theoretical prediction.

We now look at the performance of the dynamic equations. The dynamic prediction is more easily affected by noise than the final size prediction, so we use larger population sizes. We again take the degree distribution of table 1. We begin with 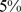 infected, either randomly chosen, or chosen as before proportional to the square of the degree. A comparison of individual simulations with theoretical predictions is in figure 5. The theory accurately predicts the dynamics of epidemics in large populations, but there is significant stochasticity in smaller populations. The theory is able to capture the impact of the biased selection of initial infections.

### Intervention Impact

We can use our equations to compare the impact of several interventions. We consider an epidemic spreading in the population, and at some intermediate time we introduce a change in the disease or population. Because the system changes at a time with a non-negligible amount of infection in the population, the equations derived assuming a negligible proportion infected fail.

Consider a population in which 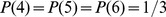. Assume we initially infect a small, randomly chosen proportion of the population, 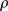 at 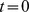. Thus we have 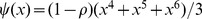, 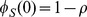, 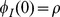, and 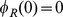. We take 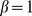 and 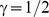.

We consider three interventions that may be introduced at time 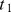. All are aimed at “halving” the transmission rate, but they do this in different ways. In mass-action based models, these would all have the same effect. We can clearly identify differences using our approach.

- An intervention that reduces 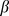 by a factor of 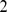.
- An intervention that reduces 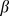 so that per-contact transmission probability 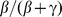 is reduced by a factor of 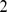.
- An intervention that eliminates half of the partnerships randomly.

The distinction between the first two comes from the fact that partnerships have duration, so reducing 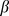 by a factor of 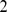 does not reduce the infection probability by a factor of 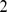. The expected number of transmissions an individual sends to a given partner is 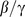, but once the partner is infected, the subsequent transmissions are irrelevant. If we use mass action assumptions however, each transmission is to a replacement partner. In each case, halving 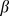 halves the total number of transmissions, but when partnerships have nonzero duration, a larger proportion of transmissions have no effect. The probability of transmitting at least once along a static partnership is 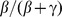. So to reduce infection probability by a given factor requires a larger reduction to 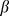. Note that the work of [24] suggests that in Configuration Model networks the final size of our second and third intervention will be the same, (but that in clustered networks it will be different).

We will demonstrate our approach in all three cases, restarting the calculations when the intervention is put into place. In all cases, this allows us to use the conditions at 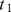 to predict the final size. We take 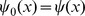, 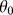, 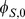, 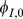, and 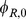 to correspond to time less than 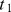. We use a subscript of 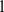 for times after 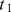. We solve the original equations, and then use the results to initialize the second set of variables.

#### Case 1

We begin by reducing 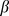 by a factor of 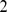 at time 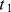. Until time 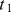, we are solving the original equations. By solving the original system until 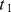 we have 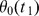. The probability an individual of degree 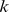 is susceptible at time 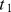 is 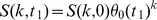. So our new 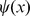 is 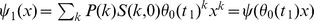. We take our new 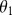 to have 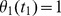. The intervention we are doing has no impact on the probability a partner is in any given state. 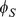, 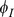, and 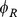 keep the same proportion, but are scaled up to sum to 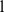 so each is scaled by 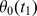. For example, 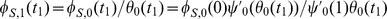.

We restart the solutions with these new values.

#### Case 2

The total probability of transmitting to a partner is 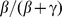. For this intervention we change 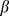 so that 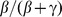 is reduced by a factor of 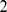 at time 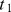. This proceeds exactly as above except that the new value of 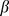 must be smaller.

#### Case 3

When we delete half the edges at random, we do not affect the probability that a random partner is in any given state. So the 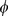 variables rescale in the same way as for changing 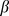 in the previous cases. However, 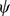 undergoes a more significant change. As a starting point, consider 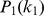, the probability an individual has degree 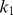 after edges are deleted. This depends on 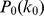, the probability of having 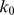 edges prior to deletion. The relation is
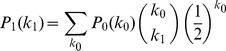



The probability the individual has degree 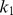 and is susceptible at time 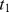 is
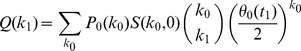



So if we restart the calculations at 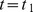 we have 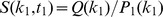 and
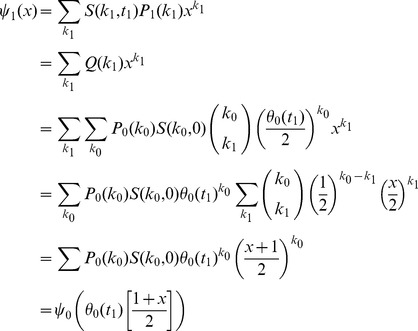



(in general if we delete edges with probability 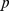 and keep with probability 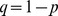, then the new function is 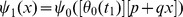). Using this new 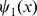, the same system of equations holds.

**Figure 6:**
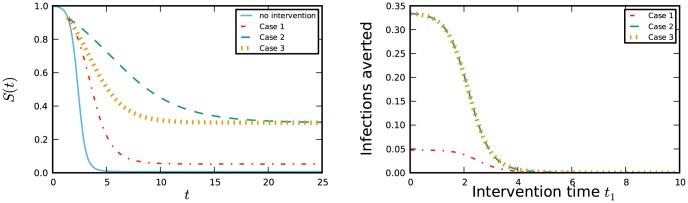
The impact of interventions. Epidemics begin at 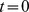 with 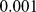 of the population infected. Left: epidemic curve without interventions, and with each intervention introduced at time 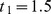. Right: horizontal axis is 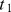, showing final effectiveness if interventions introduced at different times

Figure 6 compares these strategies. As anticipated, the final sizes resulting from cases 2 and 3 are identical, regardless of the time of intervention (indeed this is straightforward to show from the final size relation). However, we see that the dynamics are significantly different.

There are other ways we could capture these interventions mathematically. We note that in both case 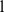 and case 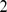, we could also capture the intervention by simply using a step change in 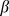. Case 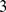 could be captured by reducing 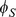, 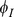 and 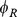 each by half and placing the other half into a new inactive compartment 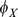 for the deleted edges. However, each of these is *ad hoc* and dependent on the precise details of the case. Using the approach presented above, we have a standard approach that will apply across a wide range of interventions.

### Bifurcation analysis

We try to gain a better understanding of the epidemic threshold and what happens to the final size as the initial proportion infected is increased. Consider the final size relation found from
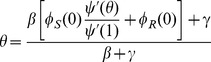



If 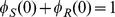, then we find that 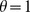 is a solution to these equations. Biologically this states that if there is no infection initially [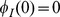] there will be no infection later. To study what happens when 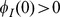 (but possibly arbitrarily small), we begin by first analyzing the structure of the dynamical equation for 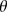 under the assumption that 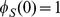 and 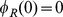, taking 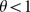. These assumptions contradict our initial conditions, but understanding the bifurcation in this system first will lead to an easier understanding of the full system with 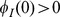.

If 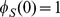 and 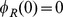, the differential equation for 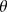 becomes
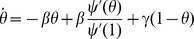
which has 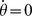 whenever
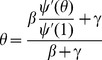



Clearly 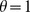 is an equilibrium. Close to 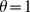, we write 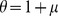, so 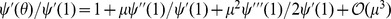. The value of 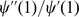 plays a key role in this analysis. Its biological interpretation comes from looking at a random infected individual's partner 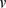 early in the epidemic. If 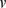 is infected by that random infected individual, then 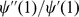 is the expected number of additional partners 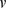 has (its *excess degree*).

Substituting into the equation for the equilibrium we have
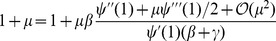
which yields
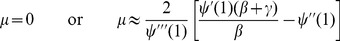
(5)


So there is a bifurcation as the bracketed term passes through zero, when 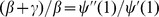. This is the well-known epidemic threshold [11]. The bifurcation is transcritical and corresponds to 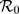 increasing through 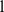. If 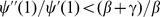 (that is 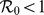) then 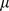 is positive and the corresponding equilibrium has 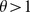 and is unstable, while the equilibrium at 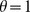 is stable. In our case, we will not observe 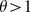 because 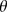 is a probability. If however 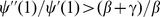 (that is 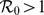), then the corresponding equilibrium has 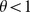 and is stable while the equilibrium at 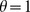 is unstable.

Previous studies found these approximate fixed points for 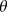, and used a slightly different definition for 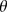 such that 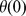 was slightly less than 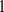. So the biologically implausible prediction is that for 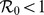 the value of 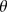 increases towards the stable fixed point at 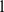. For 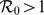 the prediction is more meaningful: 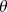 decreases away from the unstable fixed point at 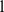 to the lower, stable fixed point. When we do a more careful analysis, we now have 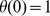 and 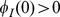. The stable fixed point that was at 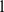 for 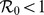 will be slightly decreased to 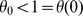. So 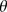 will decrease to 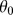 when 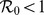. For 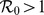 the unstable fixed point that was at 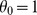 is slightly increased, so 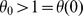. So 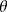 will decrease away from 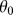. When the number of infections decays immediately (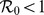) we care about a small error in the prediction caused by the fact that the initial proportion infected is not exactly zero, while if the number of infections grows (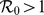), the error caused by treating the initial proportion infected as asymptotically 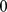 is insignificant.

**Figure 7:**
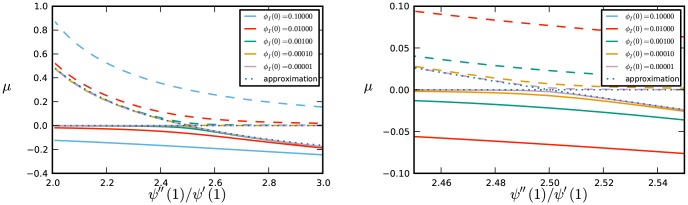
Bifurcation diagram with 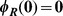, 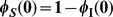, and 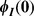 as given. The figure on right zooms in on the bifurcation point. Disease parameters are 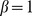 and 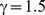. In each all members of the population have degree either 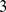 or 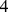, with the proportions chosen so that 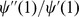 takes the values on the horizontal axis. The dashed curves denote unstable equilibria and the solid curves stable equilibria. Approximate curves (dotted) come from equation (5). Only the equilibria with 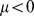 are biologically meaningful.

We are now able to consider the effect of realistic initial conditions. We keep 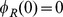, but take 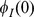 to be a small positive number with 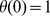 and 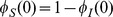. The bifurcation diagram changes slightly. Compared to the equations assuming 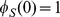, this has the effect of decreasing 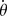 slightly, so the equilibrium values shift as shown in figure 7. In fact, strictly speaking, there is no bifurcation for 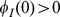.

Below the bifurcation value of 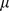, the equilibrium 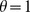 is slightly reduced to a 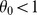, but remains stable. The solution with initial condition 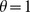 converges to this equilibrium. Above the bifurcation, the 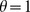 equilibrium is slightly increased to a 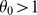 and is unstable. The other equilibrium with smaller 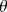 is stable and its location is decreased slightly. Thus the solution with initial condition 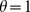 decreases and converges to the stable solution.

## Results and Discussion

In this paper we have extended previous mathematical methods for epidemic spread in networks to allow for simple models that can capture the impact of a non-negligible initial condition.

Our system of equations (1) and (2) are mathematically simple and can be solved numerically with standard tools. Changes in the population's degree distribution affect 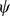, but do not otherwise alter the structure of the equations, and in particular, the population can have arbitrarily large maximum degree without requiring any increase in the number of equations.

Our modeling approach accurately predicts the size and dynamics of simulated epidemics with arbitrary sized initial conditions. The approach allows us to compare interventions introduced during the epidemic. We further see that the discrepancy found by [14] apparently results from the fact that the initial proportion infected was nonnegligible.

We are able to investigate the dynamical structure of the equilibria found in the equations, showing how the epidemic threshold is modified when a non-negligible proportion of the population is infected. This helps resolve the apparent contradiction in earlier work in which 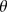, a measure of the probability of having escaped transmission, could increase in time. On biological grounds 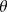 must be monotonically decreasing. The mathematical inconsistency resulted from the assumption of a small initial condition. For 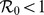, in the small initial condition limit, there is an attracting equilibrium value where the cumulative amount of infection is very small. For a reduced initial infection, the equilibrium moves, going to no infection in the limit. The previous analyses effectively froze this equilibrium at its asymptotic limit and then considered a small, but nonzero initial amount of infection. So the initial condition was on the wrong side of the equilibrium and the mathematical model attempts to return to its frozen equilibrium of no cumulative infection rather than approaching the true equilibrium with a small number of recovered individuals. When 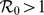, this effect does not matter because the frozen equilibrium is repelling and still on the correct side of the initial condition.

One of the most obvious applications of these results is to the understanding of the impact of an intervention that begins after a disease has established itself. This has been a weakness of network models for some time: the earliest low-dimensional models could only calculate static quantities such as the final size of epidemics assuming no intervention, while more recent approaches that calculate the dynamics [7], [15], [22] have been restricted to the assumption of asymptotically small initial conditions, again with no change in the population behavior. Because we now have a low-dimensional model that can account for large initial conditions, we can use this to restart our calculations when an intervention is to be implemented, or we can use the final size relation to quickly compare intervention effectiveness.

We have analyzed the bifurcation structure of the final size relation, and used this to explain an apparent discrepancy in earlier work if 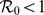. The previous models that assumed small initial condition also implicitly assume that 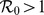. This resulted in a disturbing prediction for 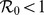 that transmissions could be reversed as time progresses, and infected individuals are uninfected. Once we correctly account for the initial condition this apparent discrepancy disappears.

If variance is large enough, then there may be a small number of very high degree individuals who have a macroscopic effect on the dynamics. Usually, increasing the population size will “drown out” the effects of individuals. However, when the variance is sufficiently large increasing the population size results in a small number of much higher degree individuals. These again have a macroscopic effect on the dynamics. Deterministic predictions will not be accurate: for example, how long the highest degree individual remains infected will influence the final size. The work of [8] rigorously studied the equations using small initial conditions, and showed that if all moments up to the fifth moment were finite, then these equations are accurate in the limit of a large network. More recently [25] has shown that the equations remain valid with much weaker assumptions. The equations will fail if the degree distribution is too broad exactly because these very rare individuals are able to have a population-scale impact on the dynamics, so the specific time and duration of their infections matter.

We expect to be able to recover from this challenge however. After the epidemic has run for a short period of time in a Configuration Model network, all of these high degree individuals have been infected and recovered. The remaining population will have significantly reduced moments. At this point, the stochastic effects are “frozen in”: the dynamics are now deterministic. We can use the observed conditions at this time to initialize our new system of equations.

There are some assumptions implicit in our derivation that deserve further attention. The model fails if 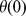, 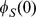, 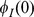, or 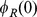 depend on degree of 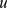. So if for example, we select high degree individuals and then infect their partners (leaving the high degree individuals uninfected), the model will not account for the fact that higher degree individuals are more likely to have infected partners at 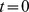. The approach will fail. This is discussed in more detail in [26].

To be clear, the model does not fail if the initial individuals infected have higher (or lower) degree. This simply affects the initial conditions. Indeed, we expect that if the infection is initially spreading stochastically in the population, and we set 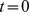 to be when enough cases are infected to have deterministic behavior, we will see that at 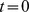 a disproportionate number of higher degree individuals have been infected. So 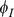 and 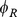 may initially be larger than 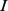 and 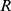.

We finally note that in [15], [16], a number of generalizations of the EBCM equations were considered. The basic approach we have used here can be applied to any of those generalizations.
